# L type Ca^2+ ^channel blockers prevent oxaliplatin-induced cold hyperalgesia and TRPM8 overexpression in rats

**DOI:** 10.1186/1744-8069-8-7

**Published:** 2012-01-31

**Authors:** Takehiro Kawashiri, Nobuaki Egashira, Kentaro Kurobe, Kuniaki Tsutsumi, Yuji Yamashita, Soichiro Ushio, Takahisa Yano, Ryozo Oishi

**Affiliations:** 1 Department of Pharmacy, Kyushu University Hospital, 3-1-1 Maidashi, Higashi-ku, Fukuoka 812-8582, Japan

## Abstract

**Background:**

Oxaliplatin is an important drug used in the treatment of colorectal cancer. However, it frequently causes severe acute and chronic peripheral neuropathies. We recently reported that repeated administration of oxaliplatin induced cold hyperalgesia in the early phase and mechanical allodynia in the late phase in rats, and that oxalate derived from oxaliplatin is involved in the cold hyperalgesia. In the present study, we examined the effects of Ca^2+ ^channel blockers on oxaliplatin-induced cold hyperalgesia in rats.

**Methods:**

Cold hyperalgesia was assessed by the acetone test. Oxaliplatin (4 mg/kg), sodium oxalate (1.3 mg/kg) or vehicle was injected i.p. on days 1 and 2. Ca^2+ ^(diltiazem, nifedipine and ethosuximide) and Na^+ ^(mexiletine) channel blockers were administered p.o. simultaneously with oxaliplatin or oxalate on days 1 and 2.

**Results:**

Oxaliplatin (4 mg/kg) induced cold hyperalgesia and increased in the transient receptor potential melastatin 8 (TRPM8) mRNA levels in the dorsal root ganglia (DRG). Furthermore, oxalate (1.3 mg/kg) significantly induced the increase in TRPM8 protein in the DRG. Treatment with oxaliplatin and oxalate (500 μM for each) also increased the TRPM8 mRNA levels and induced Ca^2+ ^influx and nuclear factor of activated T-cell (NFAT) nuclear translocation in cultured DRG cells. These changes induced by oxalate were inhibited by nifedipine, diltiazem and mexiletine. Interestingly, co-administration with nifedipine, diltiazem or mexiletine prevented the oxaliplatin-induced cold hyperalgesia and increase in the TRPM8 mRNA levels in the DRG.

**Conclusions:**

These data suggest that the L type Ca^2+ ^channels/NFAT/TRPM8 pathway is a downstream mediator for oxaliplatin-induced cold hyperalgesia, and that Ca^2+ ^channel blockers have prophylactic potential for acute neuropathy.

## Background

Oxaliplatin, a platinum-based chemotherapeutic agent, is widely used for treatment of colorectal cancer. However, oxaliplatin frequently causes severe acute and chronic peripheral neuropathies. Acute neuropathy is peculiar to oxaliplatin and includes acral paresthesias enhanced by exposure to cold [1-4]; the acute neuropathy is not attributed to morphological damage to the nerve [5,6]. On the other hand, the chronic neuropathy is characterized by loss of sensory and motor function after long-term oxaliplatin treatment, and it is similar to cisplatin-induced neurological symptoms [4]. Recently, we reported that repeated administration of oxaliplatin induced cold hyperalgesia in the early phase and mechanical allodynia in the late phase in rats [7]. Oxaliplatin is metabolized to oxalate and dichloro (1,2-diaminocyclohexane)platinum [Pt(dach)Cl_2_] [8]. We reported that oxalate and platinum metabolites were involved in the cold hyperalgesia and mechanical allodynia, respectively, and that intravenous pre-administration of Ca^2+ ^or Mg^2+ ^prevented the cold hyperalgesia but not mechanical allodynia in rats [7].

Oxaliplatin-induced acute neuropathy is termed a 'channelopathy', as oxaliplatin and oxalate were shown to modulate voltage-gated Na^+ ^and K^+ ^channels in several types of neurons [9-12]. For example, oxaliplatin increases the amplitude and duration of compound action potentials interacting with voltage-gated Na^+ ^channels in rat sensory neurons [9]. Furthermore, oxaliplatin prolongs the duration of the A-fiber compound action potential related to K^+ ^channels [12]. Thus, the effect of oxaliplatin on Na^+ ^and K^+ ^channels is thought to be involved in acute neuropathy [13].

Transient receptor potential (TRP) melastatin 8 (TRPM8), an ion channel that belongs to the TRP family, is activated by innocuous cold (< 25°C) or menthol [14,15]. Recently, an increase in TRPM8 mRNA levels was reported to be involved in the oxaliplatin-induced cold hyperalgesia in mice [16]. However, the molecular mechanisms mediating the acute neuropathy remain unclear. In the present study, we investigated the involvement of voltage-gated Ca^2+ ^channels, nuclear factor of activated T-cell (NFAT) and TRPM8 in the oxaliplatin-induced cold hyperalgesia, as up-regulation of TRP channel 1 (TRPC1) expression is induced by store-operated calcium (SOC) channel/NFAT, a transcription factor regulated by the calcium signaling pathway [17]. Furthermore, we investigated the effects of Ca^2+ ^channel blockers on oxaliplatin-induced cold hyperalgesia in rats.

## Methods

### Drugs and chemicals

Oxaliplatin (Elplat^®^) was obtained from Yakult Co., Ltd. (Tokyo, Japan). Sodium oxalate was provided by Wako Pure Chemical Industries, Ltd. (Osaka, Japan). Mexiletine hydrochloride, nifedipine, diltiazem hydrochloride and ethosuximide were purchased from Sigma-Aldrich, Co. (MO, USA). Vivit was purchased from Calbiochem (Darmstadt, Germany).

### Animals

Male Sprague-Dawley rats weighing 200-250 g (Kyudo Co., Saga, Japan) were used. Rats were housed in groups of four to five per cage with lights on from 07:00 to 19:00 h. Animals had free access to food and water in their home cages. All experiments were approved by the Experimental Animal Care and Use Committee of Kyushu University according to the National Institutes of Health guidelines, and we followed the International Association for the Study of Pain (IASP) Committee for Research and Ethical Issues guidelines for animal research [18].

### Behavioral test

Cold hyperalgesia was assessed by the acetone test. Rats were placed in a clear plastic box (20 × 17 × 13 cm) with a wire mesh floor, and were allowed to habituate for 30 min prior to testing. Fifty microliters of acetone (Wako Pure Chemical Industries, Ltd.) was sprayed onto the plantar skin of each hind paw 3 times with a Micro Sprayer^® ^(Penn Century Inc., PA, USA), and the number of withdrawal response was counted for 40 s from the start of the acetone spray. Acetone tests were performed on days 0 (pre), 3, 5, 8 and 15. Oxaliplatin and sodium oxalate were dissolved in 5% glucose solution. Oxaliplatin (4 mg/kg), sodium oxalate (1.3 mg/kg) or vehicle was injected i.p. on days 1 and 2. The vehicle-treated rats were injected with 5% glucose solution. Mexiletine hydrochloride, diltiazem and ethosuximide were dissolved in 5% saline. Nifedipine was suspended in 1% carboxymethylcellulose sodium solution. Mexiletine, diltiazem, nifedipine and ethosuximide were administered p.o. simultaneously with oxaliplatin or oxalate on days 1 and 2.

### Cell cultures

Male Sprague-Dawley rats (6 weeks old, Kyudo Co.) were anesthetized with sodium pentobarbital, and the L 4-6 dorsal root ganglia (DRG) cells were removed and primary cultured. Ganglia were incubated with 0.125% (w/v) collagenase type 1 (Worthington Biochemical Corp., NJ, USA) at 37°C for 90 min followed by incubation with 0.25% (w/v) trypsin-EDTA (Gibco BRL, CA, USA) for 30 min. DRG cells were grown in Dulbecco's modified Eagle's medium (MP Biomedicals, Inc., CA, USA) with 2 mM L-glutamine and 10% FBS. The cells were cultured at 37°C in air supplemented with 5% CO_2 _under humidified conditions. Oxaliplatin, oxalate, mexiletine, diltiazem and ethosuximide were dissolved in medium. Nifedipine and vivit were dissolved in 0.2% DMSO.

### Measurement of intracellular Ca^2+ ^level

Cells were loaded with 5 μg/mL of Fura-2/AM (Dojindo Lab., Kumamoto, Japan) and then incubated for 1 h at 37°C in HEPES buffer. The Fura-2/AM-loaded cells were washed and placed in HEPES buffer. The intracellular Ca^2+ ^levels were determined by emission fluorescence at 510 nm with excitation at 340 nm and 380 nm, using FlexStation3 (Molecular Devices, Inc., CA, USA).

### Immunostaining of NFATc4

Immunofluorescent staining for NFATc4 was performed using a rabbit monoclonal antibody (Cell Signaling Technology, Inc., MA, USA). Briefly, cells were cultured on cover slips, and the cover slips then rinsed with ice-cold phosphate-buffered saline and fixed with 4% (w/v) ice-cold paraformaldehyde for 30 min at -20°C. The NFATc4 antibody was diluted (1:100) with phosphate-buffered saline (PBS) containing 5% (w/v) bovine serum albumin and 0.1% Triton X-100. Cells were incubated with diluted antibody solution overnight in a humidified chamber at 4°C. After washing with PBS, cover slips were incubated at room temperature for 1 h with goat anti-rabbit IgG (1:500 dilution in PBS) that was conjugated with Alexa Fluor^® ^488 (Cell Signaling Technology, Inc.). The nucleus was stained with 4',6-Diamidino-2-phenylindole dihydrochloride (DAPI; Dojindo Lab.). NFATc4 and nuclear staining were visualized with a fluorescence microscope (BX51; Olympus Corp., Tokyo, Japan). The nuclear translocation of NFATc4 was calculated by comparing the ratio of nuclear NFATc4 immunofluorescence/total NFATc4 immunofluorescence using analysis software (Image J 1.36; Wayne Rasband, National Institutes of Health, MD, USA).

### Reverse transcription-polymerase chain reaction (RT-PCR)

mRNA was isolated from L4-6 DRG using PolyATtract^® ^System 1000 (Promega, Corp., WI, USA). cDNA was synthesized with PrimeScript^® ^1st strand cDNA Synthesis Kit (TaKaRa Bio, Inc., Shiga, Japan). PCR was performed with Gene Taq (Nippon Gene, Co., Ltd., Tokyo, Japan). The oligonucleotide primers for TRPM8 were designed based on the sequences described by Ta and colleagues [19]. The sequences of PCR primers were as follows: TRPM8, 5'-GCC CAG TGA TGT GGA CAG TA-3' (sense), 5'-GGA CTC ATT TCC CGA GAA GG-3' (antisense); glyceraldehyde-3-phosphate dehydrogenase (G3PDH), 5'-YGC CTG CTT CAC CAC CTT-3' (sense), 5'-TGC MTC CTG CAC CAC CAA CT-3' (antisense) (Sigma-Aldrich, Co.). Reactions were run for 35 cycles with 95°C denaturing cycle (30 s), 62°C annealing cycle (1 min) and 72°C extension cycle (20 s) for TRPM8, or for 30 cycles with 94°C denaturing cycle (45 s), 53°C annealing cycle (45 s) and 72°C extension cycle (1.5 min) for G3PDH. PCR products were resolved by electrophoresis on a 4% agarose gel, and the DNA was visualized by staining with ethidium bromide under ultraviolet irradiation. The intensities of the PCR products were semi-quantified densitometrically using Alpha Imager 2200 (Cell Biosciences, Inc., California, USA).

### Western blotting

The L4-6 DRG was quickly removed on day 5. The tissues were homogenized in a solubilization buffer containing 20 mM Tris-HCl (pH7.4, 2 mM EDTA, 0.5 mM EGTA, 10 mM NaF, 1 mM Na_3_VO_4_, 1 mM PMSF, 0.32 M Sucrose, 2 mg/ml aprotinine, 2 mg/ml leupeptin), and the homogenates were subjected to 4% SDS-PAGE, and proteins were transferred electrophoretically to PVDF membranes. The membranes were blocked in Tris-buffered saline Tween-20 (TBST) containing 5% BSA (Sigma-Aldrich) for an additional 1 h at room temperature with agitation. The membrane was incubated overnight at 4°C with rabbit polyclonal TRPM8 antibody (Abcam, MA, USA) and then incubated for 1 h with anti-rabbit IgG horseradish peroxidase (Jackson Immuno Research Laboratories, Inc., PA, USA). The immunoreactivity was detected using Enhanced Chemiluminescence (Perkin Elmer, Massachusetts, USA).

### Statistical analyses

Data are expressed as the mean ± SEM. Data were analyzed by the Student's *t*-test or one-way analysis of variance (ANOVA) followed by the Tukey-Kramer post hoc test to determine differences between the groups. A probability level of *p *< 0.05 was accepted as statistically significant.

## Results

### Oxaliplatin increases cold hyperalgesia and TRPM8 expression in the DRG in rats

**Figure 1:**
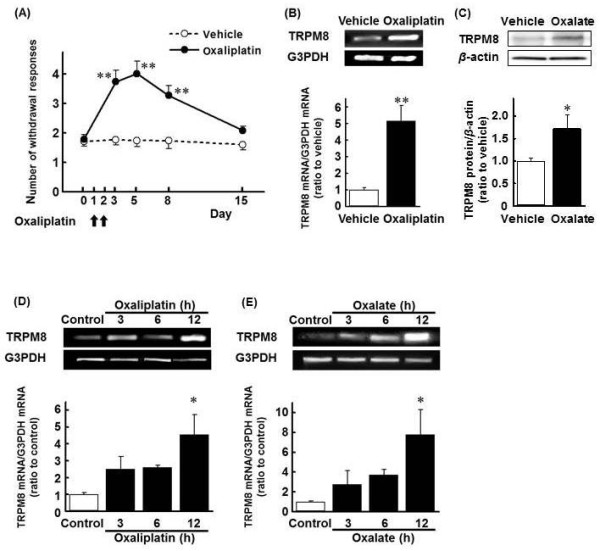
**The incidence of cold hyperalgesia (A) and expression of TRPM8 (B-E) following oxaliplatin or sodium oxalate treatment**. Oxaliplatin (4 mg/kg) or sodium oxalate (1.3 mg/kg) was administered i.p. on days 1 and 2. **A**: The acetone test was performed on days 0, 3, 5, 8 and 15. **B**: On day 5 the rat L4-6 DRG treated with oxaliplatin was harvested and the mRNA expression of TRPM8 and G3PDH were determined by PCR. **C**: On day 5 the rat L4-6 DRG treated with sodium oxalate was harvested and the protein of TRPM8 and *β*-actin were determined by Western boltting. **D**, **E**: 500 μM of oxaliplatin (D) or sodium oxalate (E) was administered to cultured DRG cells for 3, 6 or 12 h. mRNA expression of TRPM8 and G3PDH was determined by PCR. Values are expressed as the mean ± SEM of 4-6 animals (A, B) or 4-6 wells (C, D). *p < 0.05, **p < 0.01 compared with vehicle or control group.

Administration of oxaliplatin (4 mg/kg, p.o., on days 1 and 2) significantly increased the number of withdrawal responses to cold stimulation by acetone spray in rats (Figure 1A, days 3, 5 and 8: *p *< 0.01). This increase in withdrawal response had disappeared on day 15. On day 5, TRPM8 mRNA levels in the L4-6 DRG of oxaliplatin-treated rats markedly increased as compared with those of vehicle-treated rats (Figure 1B, *p *< 0.01). Also, oxalate treatment significantly induced the increase in TRPM8 protein in the L4-6 DRG (Figure 1C, *p *< 0.05).

### Oxaliplatin and oxalate increase the TRPM8 mRNA levels in primary cultured DRG cells

Treatment with either oxaliplatin (Figure 1D) or oxalate (Figure 1E) for 12 h markedly increased the TRPM8 mRNA levels in primary cultured DRG cells (*p *< 0.05 for both).

### Oxaliplatin and oxalate increase the intracellular Ca^2+ ^levels in primary cultured DRG cells

**Figure 2:**
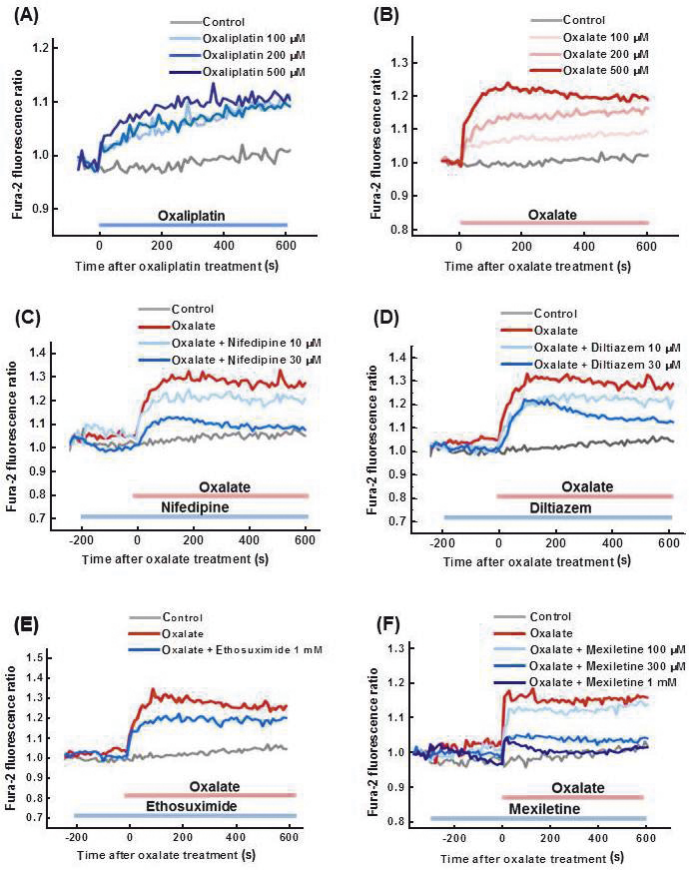
**Increase in the intracellular Ca^2+ ^following oxaliplatin or oxalate treatment in primary cultured DRG cells**. Oxaliplatin (**A**: 100-500 μM) or sodium oxalate (**B**: 100-500 μM) was administered to cultured DRG cells. Nifedipine (**C**: 10-30 μM), diltiazem (**D**: 10-30 μM), ethosuximide (**E**: 1 mM) or mexiletine (**F**: 100 μ-1 mM) were co-administered with sodium oxalate (500 μM) to cells. Intracellular Ca^2+ ^levels were determined based on Fura-2 fluorescence (340 nm/380 nm). Values are expressed as the mean of 4-8 wells.

Oxaliplatin and oxalate (100-500 μM) induced dose-dependent increases in intracellular Ca^2+ ^levels in cultured DRG cells (Figure 2A, B). The percentages of DRG neurons that responded to oxaliplatin and oxalate were 69.2% and 64.0%, respectively. Nifedipine (30 μM), an L type Ca^2+ ^channel blocker, and diltiazem (30 μM), an L/T type Ca^2+ ^channel blocker, inhibited the increase in intracellular Ca^2+ ^levels induced by oxalate (500 μM) (Figure 2C, D). Mexiletine, a Na^+ ^channel blocker, also dose-dependently inhibited the oxalate-induced increase in intracellular Ca^2+ ^levels (Figure 2F). By contrast, ethosuximide (1 mM), a T type Ca^2+ ^channel blocker, only weakly attenuated the oxalate-induced increase in intracellular Ca^2+ ^levels (Figure 2E).

### Oxaliplatin and oxalate induce NFAT nuclear translocation in primary cultured DRG cells

**Figure 3:**
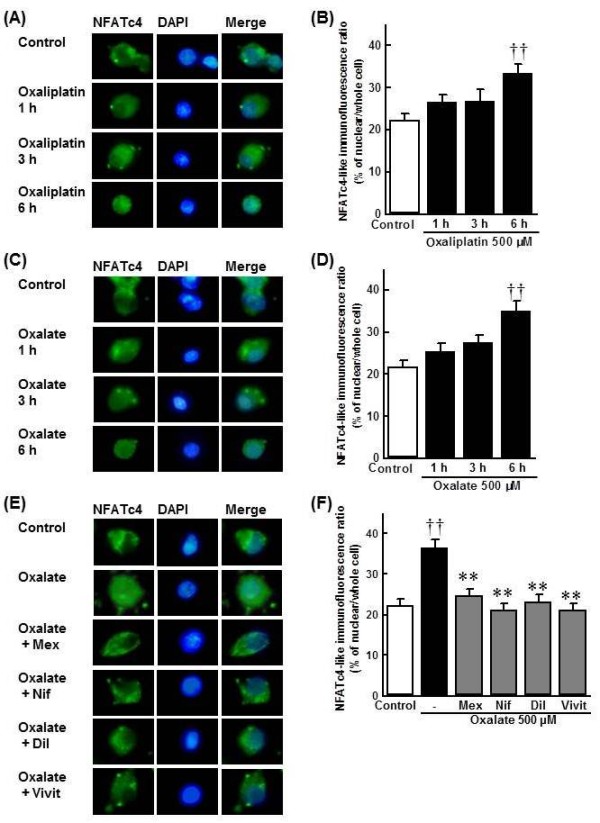
**NFAT nuclear translocation in primary cultured DRG cells**. Oxaliplatin (**A**, **B**: 500 μM for 1-6 h) or sodium oxalate (**C**, **D**: 500 μM for 1-6 h) was administered to cultured DRG cells. **E**, **F**: Mexiletine (Mex, 1 mM), nifedipine (Nif, 30 μM), diltiazem (Dil, 30 μM) or vivit (2 μM) was co-administered with sodium oxalate (500 μM) to cells for 6 h. NFATc4 immunostaining (green) and nuclear staining with DAPI (blue). NFATc4 and DAPI-positive nuclei were visualized by fluorescence microscopy (A, C, E). The nuclear translocation of NFATc4 was calculated by comparing the ratio of nuclear NFATc4 immunofluorescence/total NFATc4 immunofluorescence (B, D, F). Values are expressed as the mean ± SEM of 24-33 cells. ††*p *< 0.01 compared with control group, **p < 0.01 compared with oxalate group.

Treatment with oxaliplatin (500 μM) for 6 h induced NFAT nuclear translocation (Figure 3A, B, 6 h: *p *< 0.01). Similarly, 500 μM oxalate caused NFAT nuclear translocation (Figure 3C, D, 6 h: *p *< 0.01). Mexiletine (1 mM), nifedipine (30 μM) and diltiazem (30 μM) completely blocked the oxalate-induced NFAT nuclear translocation (500 μM) (Figure 3E, F, *p *< 0.01). Similarly, vivit (2 μM), a selective NFAT inhibitor, completely blocked the oxalate-induced NFAT nuclear translocation (Figure 3E, F, *p *< 0.01).

### Ca^2+ ^and Na^+ ^channel blockers inhibit the oxalate-induced increase of TRPM8 mRNA levels in cultured DRG cells

**Figure 4:**
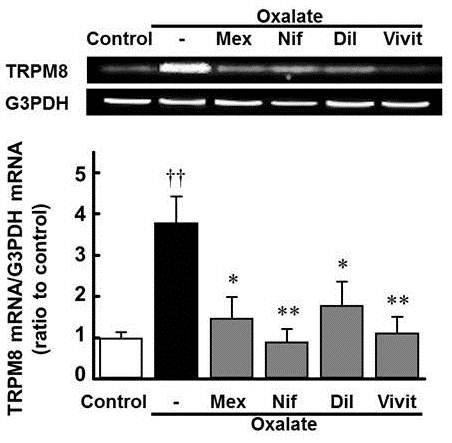
**Reversal of the oxalate-induced increase in TRPM8 mRNA in cultured DRG cells by Ca^2+ ^and Na^+ ^channel blockers**. Mexiletine (Mex, 1 mM), nifedipine (Nif, 30 μM), diltiazem (Dil, 30 μM) or vivit (2 μM) was co-administered with sodium oxalate (500 μM) to cells for 12 h. The mRNA expression of TRPM8 and G3PDH were determined by PCR. Values are expressed as the mean ± SEM of 6 wells. ††*p *< 0.01 compared with control group, *p < 0.05, **p < 0.01 compared with oxalate group.

Mexiletine (1 mM), nifedipine (30 μM) and diltiazem (30 μM) reversed the increase in TRPM8 mRNA levels induced by oxalate (500 μM, 12 h) (Figure 4, mexiletine and diltiazem: *p *< 0.05; nifedipine: *p *< 0.01). Similarly, vivit (2 μM) completely reversed the oxalate-induced increase in TRPM8 mRNA levels (*p *< 0.01).

### Ca^2+ ^and Na^+ ^channel blockers inhibit the oxaliplatin-induced cold hyperalgesia and increase in TRPM8 mRNA levels in the DRG in rats

**Figure 5:**
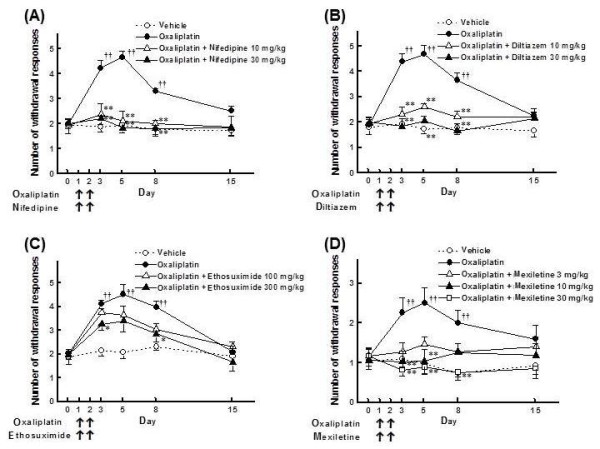
**Reversal of the oxaliplatin-induced cold hyperalgesia in rats by Ca^2+ ^and Na^+ ^channel blockers**. Oxaliplatin (4 mg/kg) was administered i.p. on days 1 and 2. Nifedipine (**A***: *10 and 30 mg/kg), diltiazem (**B**: 10 and 30 mg/kg), ethosuximide (**C**: 100 and 300 mg/kg) or mexiletine (**D**: 3-30 mg/kg) was orally co-administered with oxaliplatin. Acetone test was performed on days 0, 3, 5, 8 and 15. Values are expressed as the mean ± SEM of 6-10 animals. ††*p *< 0.01 compared with vehicle group, *p < 0.05, **p < 0.01 compared with oxaliplatin group.

**Figure 6:**
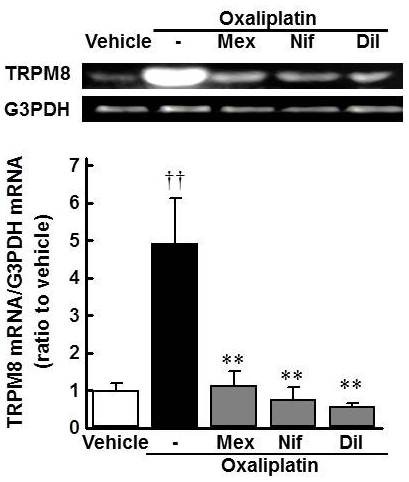
**Reversal of the oxaliplatin-induced increase of TRPM8 mRNA in rat DRG neurons by Ca^2+ ^and Na^+ ^channel blockers**. Oxaliplatin (4 mg/kg) was administered i.p. on days 1 and 2. Mexiletine (Mex, 30 mg/kg), nifedipine (Nif, 30 mg/kg) or diltiazem (Dil, 30 mg/kg) was orally co-administered with oxaliplatin. The expression of TRPM8 and G3PDH mRNAs were determined by PCR on day 5. Values are expressed as the mean ± SEM of 5 animals. ††*p *< 0.01 compared with vehicle group, **p < 0.01 compared with oxaliplatin group.

Co-administration with nifedipine (10, 30 mg/kg, p.o.) completely inhibited the oxaliplatin-induced increase in withdrawal responses to acetone spray in rats (Figure 5A, *p *< 0.01). Diltiazem (10, 30 mg/kg, p.o.) also strongly inhibited the oxaliplatin-induced increase in withdrawal responses (Figure 5B, *p *< 0.01). Similarly, mexiletine (10, 30 mg/kg, p.o.) attenuated the oxaliplatin-induced increase in withdrawal responses (Figure 5D, *p *< 0.01). By contrast, ethosuximide (300 mg/kg, p.o.) only weakly prevented the oxaliplatin-induced increase in withdrawal responses (Figure 5C, days 3 and 8: *p *< 0.05). Moreover, co-administration with mexiletine (30 mg/kg, p.o.), nifedipine (30 mg/kg, p.o.) or diltiazem (30 mg/kg, p.o.) completely inhibited the oxaliplatin-induced increase in TRPM8 mRNA levels on day 5 (Figure 6, *p *< 0.01).

## Discussion

Oxaliplatin was previously reported to induce cold allodynia and increase in TRPM8 mRNA levels in the DRG after 3 days in mice [16] and increase the TRPM8 mRNA levels in cultured rat DRG cells [19]. Consistent with these reports, in the present study we demonstrated that oxaliplatin induced cold hyperalgesia in rats on days 3, 5 and 8 and increased the TRPM8 mRNA levels in the DRG on day 5, the peak of cold hyperalgesia. Furthermore, we found that oxalate significantly induced the increase in TRPM8 protein in the DRG on day 5. In addition, we confirmed that oxaliplatin markedly increased the TRPM8 mRNA levels in primary cultured DRG cells.

TRPM8 is known to be involved in cold sensitivity [20] and cold allodynia after chronic nerve injury [21]. Moreover, TRPM8-deficient mice attenuate behavioral response to cold stimulation [22,23]. Oxaliplatin-induced cold allodynia is reversed by capsazepine, a blocker of both TRPM8 and TRP vanilloid 1 (TRPV1), but not by 5'-iodoresiniferatoxin, a selective TRPV1 blocker [16]. Hence, the increase in TRPM8 expression in DRG neurons may be involved in oxaliplatin-induced cold hyperalgesia. Recently, Nassini et al. [24] have reported that oxaliplatin induces mechanical and cold allodynia via TRP ankyrin 1 (TRPA1) activation in rodents. Considering these collective findings, both up-regulation of TRPM8 and activation of TRPA1 may be involved in the cold hypersensitivity by oxaliplatin. We also found that treatment with oxalate, a metabolite of oxaliplatin, markedly increased the TRPM8 mRNA levels in primary cultured DRG cells. Furthermore, oxalate significantly induced the increase in TRPM8 protein in the DRG. Oxaliplatin is rapidly metabolized to Pt(dach)Cl_2 _in rat blood *in vitro *[25], suggesting that oxalate is immediately derived from oxaliplatin. We previously reported that oxalate induced cold hyperalgesia/allodynia but not mechanical allodynia in rats [7]. Taken together, these data suggest that oxalate may be involved in the oxaliplatin-induced increase in TRPM8 expression, resulting in cold hyperalgesia.

In the present study, both oxaliplatin and oxalate increased the intracellular Ca^2+ ^levels in primary cultured DRG cells, and the oxalate-induced increase in intracellular Ca^2+ ^level was inhibited by nifedipine (an L type Ca^2+ ^channel blocker) and diltiazem (an L/T type Ca^2+ ^channel blocker). By contrast, ethosuximide (a T type Ca^2+ ^channel blocker) only weakly attenuated the oxalate-induced increase in intracellular Ca^2+^. Thus, it is likely that oxaliplatin induces Ca^2+ ^influx via mainly L type Ca^2+ ^channels. Oxaliplatin was reported to increase the amplitude and duration of compound action potentials interacting with voltage-gated Na^+ ^channels in rat sensory neurons [9], and prolong the duration of the A-fiber compound action potential related to K^+ ^channels [12]. Thus, enhancement of action potentials via Na^+ ^or K^+ ^channels might result in Ca^2+ ^influx through L type Ca^2+ ^channels. This mechanism is supported by the present result that the Na^+ ^channel blocker mexiletine completely reversed the oxalate-induced Ca^2+ ^influx.

In general, NFAT is activated and translocated into the nucleus via Ca^2+ ^signaling [26]. In the present study, both oxaliplatin and oxalate induced the nuclear translocation of NFAT in cultured DRG cells, and the oxalate-induced NFAT nuclear translocation was completely blocked by nifedipine, diltiazem and mexiletine, as well as vivit, a selective NFAT inhibitor. Furthermore, nifedipine, diltiazem, mexiletine and vivit reversed the oxalate-induced increase in TRPM8 mRNA levels in cultured DRG cells. Taken together, these data suggest that oxalate may induce up-regulation of TRPM8 expression via NFAT activation by Ca^2+ ^influx through L/T type Ca^2+ ^channels derived from Na^+ ^channels activation. We also confirmed that co-administration with nifedipine, diltiazem or mexiletine inhibited the oxaliplatin-induced cold hyperalgesia and increase in TRPM8 mRNA levels in the DRG *in vivo *in rats. Thus, the oxaliplatin-induced cold hyperalgesia is mediated by up-regulation of TRPM8 expression via Na^+ ^and Ca^2+ ^influx.

In addition, Fajardo et al. [27] have reported that L-type Ca^2+ ^channel blockers 1,4-dihydropyridines such as nifedipine activate TRPA1-mediated currents in CHO cells in electrophysiological study. However, they reported that no signs of behavioral pain were observed following local application of nifedipine to the hind paw of mice. Because nifedipine blocks electrically evoked Ca^2+ ^transients in peripheral sensory nerves [28], it is possible that these potent inhibitory actions on L-type Ca^2+ ^channels prevent the propagation of electrical impulses at nerve terminals, despite a powerful TRPA1 activation.

## Conclusions

We demonstrated that L type Ca^2+ ^channel/NFAT/TRPM8 pathway plays a crucial role in signaling the oxaliplatin-induced cold hyperalgesia. Co-administration of L type Ca^2+ ^channel blockers inhibited the oxaliplatin-induced cold hyperalgesia. Therefore, novel strategies involving Ca^2+ ^channel blockers may be useful for prevention of oxaliplatin-induced acute neuropathy.

## Competing interests

The authors declare that they have no competing interests.

## Authors' contributions

TK, NE and RO are responsible for experimental design. TK and KK are responsible for performance of behavioral test. TK, KK, KT and YY are responsible for measurement of intracellular Ca^2+ ^level, immunostaining and PCR. KT, SU and TY are responsible for performance of Western blotting. TK, NE and RO are responsible for writing the manuscript. All authors read and approved the final manuscript.
